# Epileptic Seizures and Right-Sided Hippocampal Swelling as Presenting Symptoms of Anti-IgLON5 Disease: A Case Report and Systematic Review of the Literature

**DOI:** 10.3389/fneur.2022.800298

**Published:** 2022-05-10

**Authors:** Yaoqi Fu, Xiangting Zou, Ling Liu

**Affiliations:** Department of Neurology, West China Hospital, Sichuan University, Chengdu, China

**Keywords:** anti-IgLON5 disease, autoimmune encephalitis, systematic review, clinical manifestation, laboratory investigation, immunotherapy, radiological feature

## Abstract

**Background and Objective:**

Anti-IgLON5 disease is an uncommon neurological disorder characterized by diverse clinical manifestations. Although many relevant cases have been reported, our understanding of this disorder is still quite restricted. We present a rare case of anti-IgLON5 disease and performed a comprehensive systematic review of all published cases to expand the clinical spectrum of this disorder.

**Methods:**

We report a 61-year-old woman with an atypical presentation of epileptic seizures with abnormal signals in her right hippocampus on brain magnetic resonance imaging (MRI). A systematic review was performed of electronic databases, including PubMed, EMBASE, China National Knowledge Infrastructure (CNKI), WanFang and VIP China Science.

**Results:**

We identified 161 cases from 65 publications. With heterogeneous clinical manifestations, we found that bulbar dysfunction, sleep apnea, gait instability and neurocognitive and behavioral symptoms are the most common symptoms of anti-IgLON5 disease. Anti-IgLON5 antibodies presented a higher positive rate and titer in the serum than in the cerebrospinal fluid (CSF). Haplotype DRB1*10:01-DQB1*05:01 is highly correlated with anti-IgLON5 disease. Only 38 patients have presented distinctive MRI alterations (26.2%). Approximately half of the cases are responsive to immunosuppressive or immunomodulatory treatment.

**Conclusion:**

Anti-IgLON5 disease is characterized by various clinical manifestations and laboratory findings. Immunotherapy may be effective in treating anti-IgLON5 disease, but the results are far from satisfactory. Studies with larger sample sizes are required to improve the current understanding of this disorder.

## Introduction

First reported in 2014 (1), anti-IgLON5 disease is characterized by heterogeneous clinical manifestations. Gaig et al. (2) described the clinical features of 22 patients with anti-IgLON5 disease and summarized four major clinical phenotypes according to the initial symptoms: (1) a predominant sleep disorder characterized by a combination of non-rapid eye movement (NREM) and rapid eye movement (REM) sleep parasomnias with obstructive sleep apnea (OSA) and stridor (3); (2) a bulbar syndrome including dysphasia, dysarthria, vocal cord paresis and acute respiratory stress; (3) a syndrome resembling progressive supranuclear palsy (PSP), with abnormal oculomotor movements and an unstable gait; and (4) cognitive impairment that may be associated with chorea (4). In addition to the major symptoms described for the published clinical phenotypes, other clinical features, such as dysautonomia and seizures (5, 6), are not rare. A strong association between haplotype HLA DRB1^*^10:01-DQB1^*^05:013 and anti-IgLON5 autoantibodies was proven *in vitro* (7), which means human leukocyte antigen (HLA) typing is key to the diagnosis.

Generally, cranial magnetic resonance imaging (MRI) of patients with anti-IgLON5 disorders is unremarkable or unspecific (4). Although various cases have been reported thus far, anti-IgLON5 disease remains under recognized. Anti-IgLON5 disease can be diagnosed when anti-IgLON5 antibodies are detected either in serum or cerebrospinal fluid (CSF). However, the typical clinical features and laboratory or radiological findings may be useful to identify possible and probable cases. It is necessary to summarize and analyze all of the cases published previously to expand the clinical spectrum of anti-IgLON5 disease.

We report a case with seizures as a major symptom, presenting with a distinctive MRI change in her right hippocampus. The patient did not show any features of the clinical phenotypes defined by Gaig et al. We performed a systematic review of all of the published cases of anti-IgLON5 disease to expand the clinical spectrum of anti-IgLON5 syndrome. In addition, we aimed to evaluate the effects of immunotherapy on anti-IgLON5 disease.

## Methods

### Systematic Review

To comprehensively investigate the clinical features and the responses to immunotherapy of anti-IgLON5 diseases, we performed a systematic review by using “IgLON5,” “anti-IgLON5,” and “IgLON5 antibody” as search terms. We scrutinized the relevant studies in electronic databases, including PubMed and EMBASE, from inception to January 2022 without any language restrictions. We also searched several Chinese electronic databases, including China National Knowledge Infrastructure (CNKI), WanFang and VIP China Science, for additional relevant studies written in Chinese. All study designs were included in the review, including clinical trials and observational studies (cohorts, case reports and case series). We considered eligible studies meeting all of the following inclusion criteria: (1) IgLON5 antibody titers in either serum or cerebrospinal fluid (CSF) samples of the patients described in the studies were classified as positive; (2) detailed clinical information for each case was available. Two reviewers separately screened the titles and abstracts to identify the potentially relevant articles. The full texts of the sorted studies were reviewed carefully to identify duplicated cases.

A standardized form containing the following information was used in the data extraction phases: age at onset, sex, disease duration or follow-up length, clinical phenotypes and symptoms, CSF investigations, anti-IgLON5 antibodies in serum and CSF, HLA-alleles analysis, radiological investigations, immunotherapy and response to immunotherapy. Data extraction was performed by two researchers independently. Any disagreement was resolved by consensus and discussion with the help of a third researcher.

Clinical phenotypes were defined as previously described (8): (1) predominant sleep disorder, (2) bulbar dysfunction, (3) movement disorder, (4) cognitive impairment that may be associated with chorea, and (5) neuromuscular manifestations including fasciculations in muscles and muscle weakness or atrophy. We also identified patients with PSP-like syndromes according to the Movement Disorder Society diagnostic criteria for PSP (MDS-PSP) (9). Response to immunotherapy was measured as the number (%) of patients who had an improvement in at least one of the main symptoms or signs defined by the clinical phenotypes after first-line therapy or subsequent therapy (10). Response to first-line therapy and response to second-line therapy (due to relapse or first-line therapy failure) should be recorded separately.

### Case Report

A 61-year-old, right-handed woman was admitted to a local tertiary hospital with a chief complaint of a sudden loss of consciousness, without any presence of myoclonic twitches of the limbs, lasting for almost 20 min. She showed evident confusion and was unable to identify her relatives after she regained consciousness. Diagnosed with acute ischemic stroke, she was treated with intravenous thrombolysis. At discharge, her symptoms were completely resolved. However, intracranial lesions (abnormal signals and increased volume of the right hippocampus and a few unspecific periventricular white matter lesions) were found on her cranial magnetic resonance imaging (MRI) when she visited the neurological outpatient clinic of our hospital for follow-up. She was readmitted to the hospital for necessary investigations. She had a past history of lung adenocarcinoma and underwent lobectomy of the right lung 2 years ago. Although she did not receive any radiotherapy or chemotherapy, there was no sign of recurrence.

Neurological examination was performed, and no evidence of any gait instability, limb ataxia, dysphagia, dysarthria, cognitive impairment or abnormal oculomotor movements was seen. Lumbar puncture showed an intracranial pressure of 107 mm H_2_O, and CSF analysis showed normal protein, glucose, cell counts, and IgG synthesis rates with no oligoclonal bands. A panel of serologic and CSF tests for antibody-mediated autoimmune encephalopathy and paraneoplastic syndromes, including anti-NMDAR, anti-AMPAR1, anti-AMPAR2, anti-LGI1, anti-CASPR2, anti-GABABR, anti-DPPX, anti-IgLON5, anti-GlyRα1, anti-mGluR5, anti-D2R, anti-Neurexin3α, anti-GAD65, anti-Hu, anti-Yo, anti-Ri, anti-CV2, anti-Ma2, anti-SOX1, anti-Tr (DNER), anti-Zic4, anti-PKCγ, anti-Recoverin and anti-Titin, was performed, and serologic and CSF testing for anti-IgLON5 was positive (with a titer of 1:320 in the serum and 1:1 in the CSF). IgLON5 antibodies were screened by immunohistochemistry on frozen sections of rat brain and confirmed by cell-based assay (CBA) using HEK 293 cells transfected with IgLON5 with the help of Kindstar Medical Laboratory Co., Ltd. (Sichuan, China). Human leukocyte antigen (HLA) typing was also performed, and HLA-DQB1^*^0501 alleles but not HLA-DRB1^*^1001 alleles were identified.

**Figure 1:**
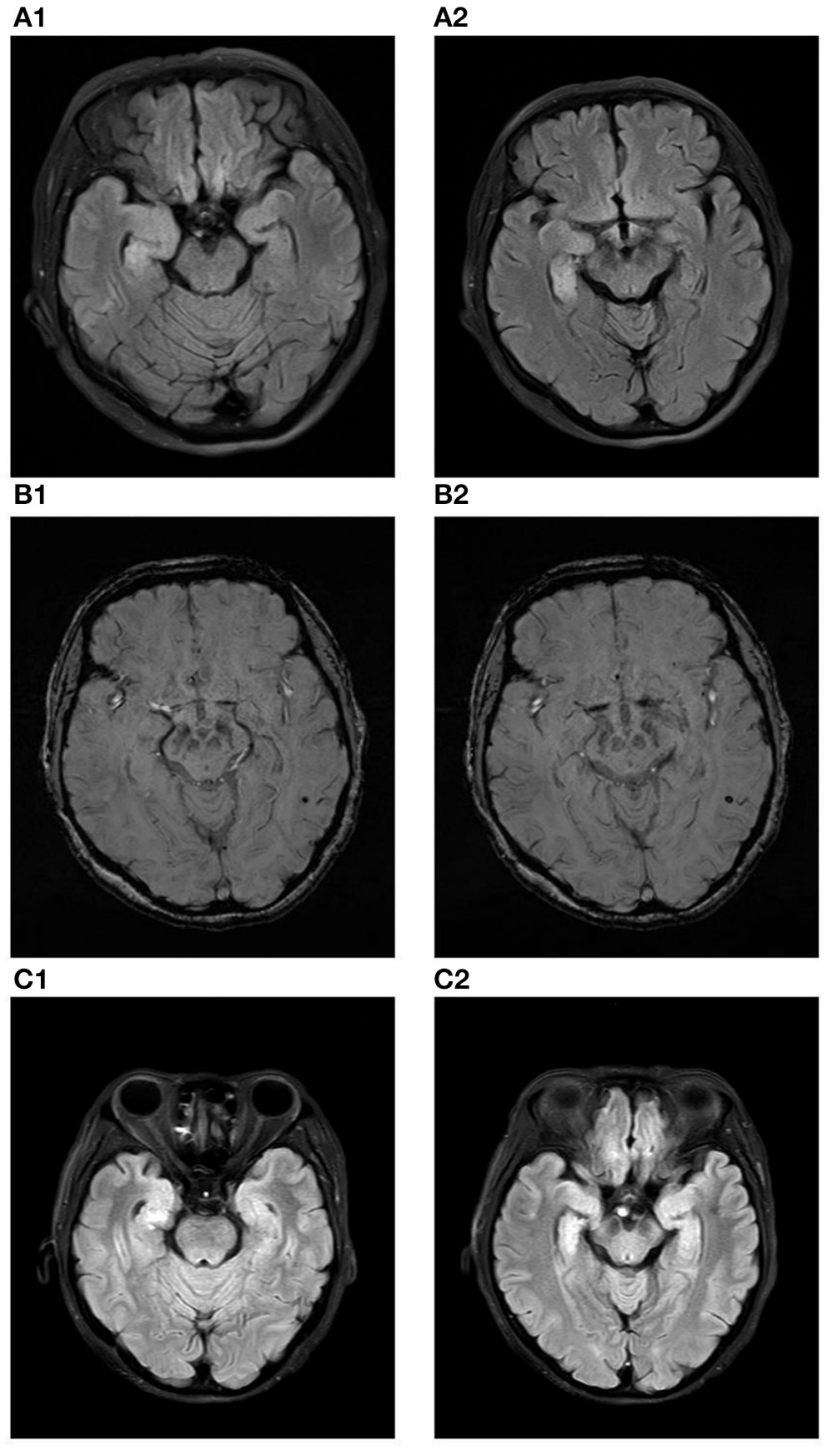
**(A1,A2)** show the increased volume of the right hippocampus and hyperintensities in the fluid attenuated inversion recovery (FLAIR) sequence at the time of initial diagnosis. **(B1,B2)** show microbleed in the left temporal lobe in the susceptibility weighted imaging (SWI) sequence at the time of initial diagnosis. **(C1,C2)** show the increased volume of the right hippocampus and hyperintensities in the fluid attenuated inversion recovery (FLAIR) sequence ten months after diagnose.

**Figure 2:**
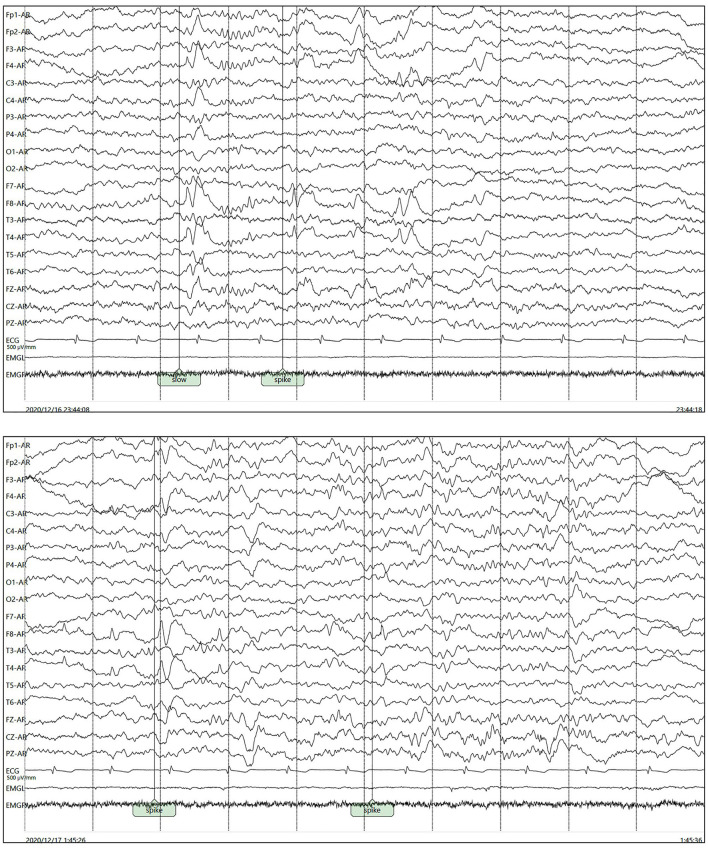
Asymmetric 3–5 Hz slow waves mixed with sharp waves and sharp-wave complexes in the right frontal pole, frontal region and anterior-middle temporal region.

Her polysomnography respiratory parameters were unremarkable. Her apnea-hypopnea index was 2.7/h (normal <5/h), and her mean oxygen saturation was 97% with a nadir of 93%. Periodic limb movements were present during sleep (12.8/h). She had poor sleep continuity, reduced sleep efficiency, poorly structured non-rapid eye movement (NREM) sleep stages and an increased rapid eye movement (REM) proportion. Her cranial MRI presented abnormal signals in the right hippocampus without enhancement as shown in Figure 1A and microbleed in the left temporal lobe as shown in Figure 1B, which was similar to the cranial MRI results at her admission. Her 24-h video electroencephalogram (EEG) showed asymmetric 3–5 Hz slow waves mixed with sharp waves and sharp-wave complexes in the right frontal region and anterior-middle temporal region as shown in Figure 2. Considering her history of lung adenocarcinoma, she underwent a whole-body 18F-flurodeoxyglucose positron emission tomography/computed tomography (^18^F-FDG PET/CT) scan. Apart from the absence of the right upper lung, no neoplastic abnormalities were found on a whole-body ^18^F-FDG PET/CT scan.

She received intravenous immunoglobulins (0.4 g/kg body weight) for 5 consecutive days and was discharged from our hospital afterward. After 3 months, she was readmitted to our department again to receive another test for antibodies related to autoimmune encephalopathy and paraneoplastic syndromes. The titer of anti-IgLON5 antibody in her serum had increased to 1:1000, but it was negative in her CSF. She kept complaining of recurrent episodes of transient unconsciousness in the morning, manifested by sudden loss of awareness of her location and a feeling of unreality, lasting approximately 1–2 s, which suggested the existence of seizures. Therefore, the anti-epileptic agent levetiracetam was added to her treatment. No recurrence of transient unconsciousness was reported since then. Seven months after her discharge, she underwent another 24-h video electroencephalogram, which was normal without any epileptiform discharges.

After 3 months, the patient developed memory impairment and recurrent episodes of confusion. She also complaint about progressive insomnia. She was readmitted into the hospital and the titer of anti-IgLON5 antibody in her serum and CSF was re-assayed. With a titer of 1:320 in the serum and 1:1 in the CSF, we considered there was a high probability of relapse of the anti-IgLON5 disease. She also underwent another cranial MRI scan, and the results was similar to the scan she underwent ten months before as shown in Figure 1C. Two courses of intravenous rituximab were administered and her symptoms completely improved after treatment.

## Results

**Figure 3:**
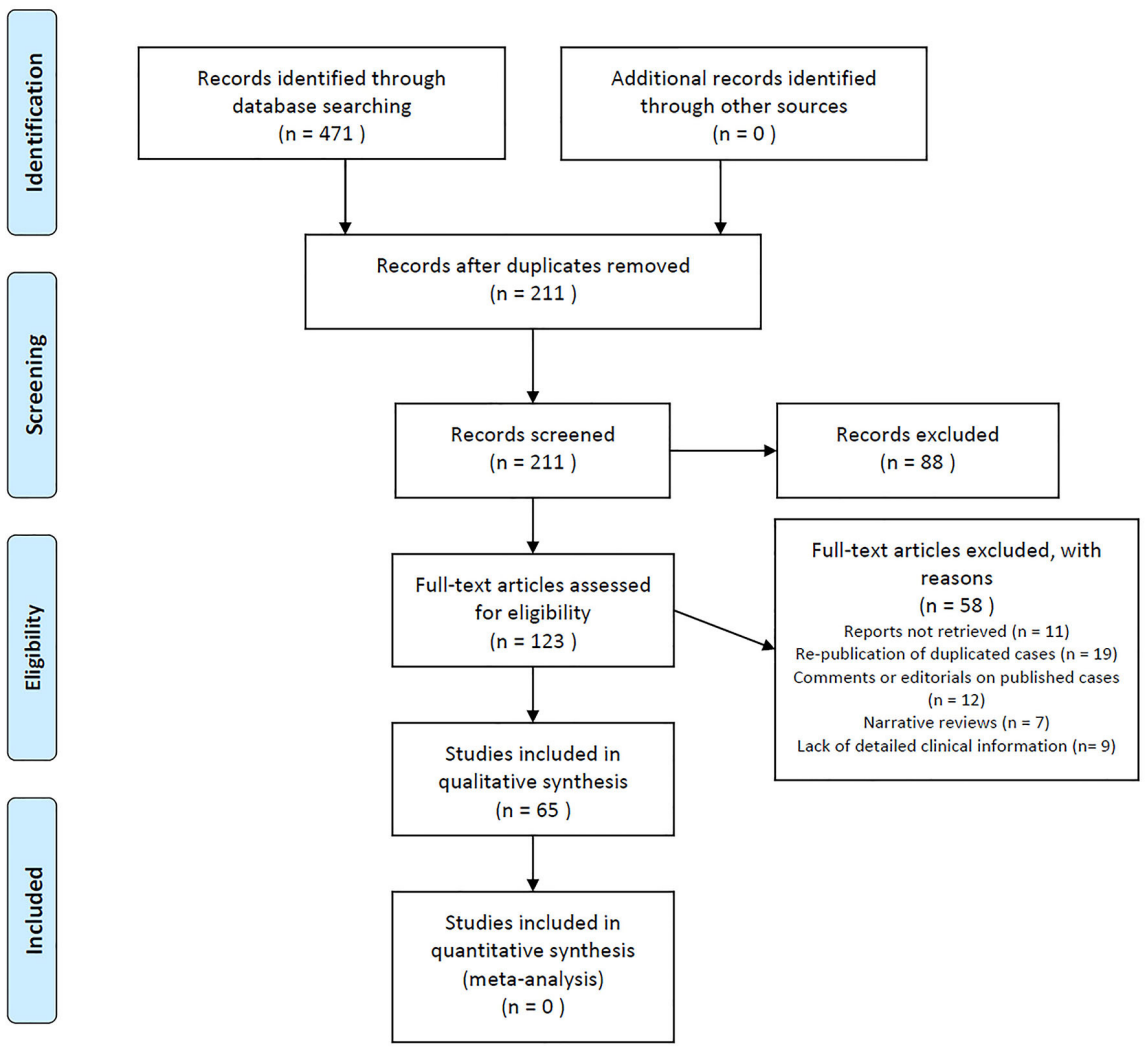
Flowchart of the literature search.

A total of 471 records were identified from searching electronic databases. After removing duplicated records, 211 citations were initially screened. By reviewing the title and abstract alone, records were removed. We identified 161 cases of anti-IgLON5 disease in 65 publications after reviewing the full text. The details of the literature selection process are shown in Figure 3. Including our case, 162 cases were included in this systematic review. Considering that the individual data could not be retrieved in the case series we included, we were unable to report the median age at onset. However, it is clear that anti-IgLON5 disease mainly affects elder adults. Only 1 pediatric patient has ever been reported (11). Among the 162 cases, 71 were women. Most of the patients presented chronic manifestations (104/136, 76.5%) The main demographic data and clinical features of the patients are shown in Table 1.

**Table 1:** Main demographic data and clinical features in described anti-IgLON5 patients.

	***N* (%) or median (range)**
Age at onset, y	(2–91)
Age at diagnosis, y	(2–91)
Male	89/160 (55.6)
Chronic presentation (in >4 months)	104/136 (76.5)
Concomitant autoimmune diseases	9/162 (5.6)
History of cancer	11/162 (6.8)
**Main complaints leading to consultation**	
Gait instability	49/162 (30.2)
Sleep disturbances	48/162 (29.6)
Movement disorders	39/162 (24.1)
Bulbar symptoms	45/162 (27.8)
Cognitive impairment	17/162 (16.7)
Other	33/162 (20.4)
**Clinical phenotypes at diagnosis**	
Movement disorders	61/162 (37.6)
Bulbar syndrome	37/162 (22.8)
Cognitive disorder	24/162 (14.8)
Sleep disorder	21/162 (13.0)
Neuromusular	9/162 (5.6)
Unclassified	10/162 (6.2)
PSP-like	22/162 (13.6)

### Clinical Manifestation

We presented all clinical symptoms of anti-IgLON5 disease at diagnosis in Table 2. Bulbar dysfunctions such as dysphagia, dysarthria and vocal cord paresis have been reported for 112 (69.1%) patients. As one of the most characteristic features of anti-IgLON5 disease, sleep disorders can manifest as abnormal sleep behaviors (72/123, 58.5%), sleep apnea (86/123, 69.9%), stridor or vocalization during sleep (41/123, 33.3%), excessive day time sleepiness (52/123, 42.3%) and insomnia (62/123, 50.4%). Regarding movement disorders, most patients presented with gait instability (94/125, 75.2%), parkinsonism (29/125, 23.2%), chorea (37/125, 29.6%) and limb ataxia (45/125, 36.0%). Parkinsonism manifests as bradykinesia with or without tremor and rigidity. Moreover, myoclonus, myokymia, dystonia, akathisia and abdominal dyskinesia were not uncommon symptoms. Ocular motor abnormalities were seen in 73 cases (45.1%). Autonomic dysfunction is also a frequently reported symptom (70/162, 43.2%), and it consists of orthostatic hypotension, intense perspiration, urinary disorders, gastrointestinal problems (constipation or diarrhea) and erectile dysfunction. Sometimes dysautonomia can be lethal, manifesting as severe bradycardia (12–14). A total of 11 patients, including the case we reported, showed epileptic seizures (10.8%) during the course of the disease. Eighty-four patients had cognitive impairment (51.9%). As for other neuropsychiatric symptoms, 16 patients had affective disorders (24.6%), 14 patients had hallucinations (21.5%), 5 patients had executive-attentive dysfunction (7.7%), 14 patients had abnormal behavior (21.5%) and 8 showed visuospatial dysfunction (12.3%). Cognitive impairment was recognized by the Mini-Mental State Examination (MMSE) or Montreal Cognitive Assessment (MoCA) in most cases.

**Table 2:** Clinical symptoms at diagnosis of anti-IgLON5 disease in described patients.

	***N* (%)**
**Sleep disturbances**	135 (83.3)
Sleep breathing problems	86/123 (69.9)
Stridor or vocalization during sleep	41/123 (33.3)
Abnormal sleep behaviors	72/123 (58.5)
Insomnia (sleep onset or fragementation)	62/123 (50.4)
excessive day sleepiness	52/123 (42.3)
**Bulbar dysfunction**	112 (69.1)
Dysphagia	88/111 (79.3)
Dysarthria	61/111 (55.0)
Episodes of acute respiratory failure or dyspnea	33/111 (29.7)
Stridor during wakefulness	14 /111(12.6)
Vocal cord palsy or laryngospasm	30/111 (27.0)
**Movement disorder**	126 (77.8)
Gait instability	94/125 (75.2)
Ataxia	45/125 (36.0)
Chorea	37/125 (29.6)
Parkinsonism	29/125 (23.2)
Dystonia	24/125 (19.2)
Abnormal body postures and rigidity	24/125 (19.2)
Tremor	24/125 (19.2)
Other hyperkinesias	41/125 (32.8)
Orofacial dyskinesia	1/41 (2.4)
Myoclonus	14/41 (34.1)
Akathisia	10/41 (24.4)
Myorhythmia	10/41 (24.4)
Myokymia	8/41 (19.5)
Abdominal dyskinesia	5/41 (12.2)
**Dysautonomia**	70 (43.2)
Urinary dysfunction (urgency and incontinence)	35/64 (54.7)
Spontaneous episodes of intense perspiration	23/64 (35.9)
Anhidrosis	3/64 (4.7)
Gastrointestinal problems (constipation, diarrhea)	11/64 (17.2)
Cardiac dysfuction	12/64 (18.8)
Orthostatic hypotension	6/64 (9.4)
Erectile dysfunction	2/64 (3.1)
**Oculomotor abnormalities**	73 (45.1)
Gaze palsies	39/73 (53.4)
Abnormal saccades	23/73 (31.5)
Abnormal pursuit	19/73 (26.0)
Nystagmus	26/73 (35.6)
Eyelid ptosis	18/73 (24.7)
Neuromuscular	21 (13.0)
Fasciculations in muscles	18/21 (85.7)
Muscle weakness/atrophy	8/21 (38.1)
**Cognitive impairment**	84 (51.9)
Dementia or memory impairment	45/62 (72.6)
Confusional episodes (delirium)	23/62 (37.1)
**Other neuropsychiatric symptoms**	
Affective disorder	16/65 (24.6)
Depression	11/16 (68.8)
Anxiety	6/16 (37.5)
Hallucination	14/65 (21.5)
Executive dysfunction	5/65 (7.7)
Abnormal behavior	14/65 (21.5)
Visuospatial dysfunction	8/65 (12.3)
**Seizure**	11/102 (10.8)

According to the clinical phenotypes defined by Gaig et al. (8), 152 patients could be classified into a certain phenotype (93.8%), as shown in Table 1. Patients with movement disorder as predominant symptom accounted for the largest proportion (61/162, 37.6%). Two manifestations, including sleep disorder (21/162, 13.0%) and cognitive impairment (24/162, 14.8%), were almost equally common. Nine patients mainly presented neuromuscular manifestations including fasciculations in muscles and muscle weakness or atrophy (5.6%), while 37 patients showed predominant bulbar symptoms (22.8%). According to the Movement Disorder Society diagnostic criteria for PSP (MDS-PSP), 22 patients could be classified as PSP-like phenotype (13.6%). What requires more attention is that 10 patients were unable to be classified into any phenotypes. As the number of reported cases increases, our understanding of this disease will improve.

### Laboratory Investigation

The titer and positive rate of the anti-IgLON5 antibodies were higher in the serum. We failed to obtain the results of anti-IgLON5 tests of the CSF in 41 patients. Anti-IgLON5 antibodies were undetectable in 12 patients (10.8%) in the CSF, as shown in Table 3. On routine CSF analysis, pleocytosis was observed in 26 patients (19.7%), and the CSF protein content was increased in 56 patients (42.2%). Intrathecal immunoglobulin and oligoclonal band synthesis were positive in 4 and 9 patients, respectively. Elevated total and phosphorylated tau were described in 2 and 4 cases, respectively. The results of CSF investigation are shown in Table 3.

**Table 3:** Laboratory investigation and radiological results at diagnosis of anti-IgLON5 disease in described patients.

	***N* (%)**
**Results of radiological investigations**	
Abnormal 18FDG-PET or SPECT	11/22 (50.0)
Abnormal DaTSCAN	3/4 (75.0)
**Brain MRI**	
Normal or non-specific changes	107/145 (73.8)
Brainstem atrophy	6/145 (4.2)
Cerebellar atrophy	5/145 (3.4)
Hippocampus lesions	5/145 (3.4)
Other abnormalities	22/145 (15.2)
**Cerebrospinal fluid investigations**	
Increased CSF protein content	56/132 (42.2)
Pleocytosis	26/132 (19.7)
Intrathecal immunoglobulin synthesis	4/16 (25.0)
Oligoclonal bands	9/46 (19.6)
Increased total tau	2/8 (25.0)
Increased phosphorylated tau	4/6 (66.7)
**Anti-IgLON5 antibodies**	
Undetectable anti-IgLON5 antibodies in CSF	12/111 (10.8)
**HLA genotyping**	
DRB1*10:01; DQB1*05:01 alleles, *n* (%)	61/105 (58.1)
Isolated DQB1*05:01, *n* (%)	7/105 (6.7)

HLA genotyping results also revealed a strong correlation between the disease and certain haplotypes. Haplotype DRB1*10:01-DQB1*05:01 was reported in 61/105 (58.1%) tested patients, isolated DQB1*05:01 in 7/105 (6.7%) and neither in 37/105 (35.2%), as shown in Table 3.

### Radiological Findings

MRI findings were reported in 145 cases. Only 38 patients presented distinctive MRI alterations (38/145, 26.2%), as shown in Table 3: six with brainstem atrophy (8) and two with lesions in the thalamus (15, 16); five with abnormal signals of the hippocampus (2, 5, 17, 18); three showed lesions in the globus pallidus (16, 19, 20) and two in the substantia nigra (16, 21); five had cerebellar atrophy (8) and one had hypothalamic lesions (22). Cortical lesions, including cortical atrophy, subcortical lesions and abnormal signals or enhancement, were present in 6 cases (23–28).

A total of 22 patients underwent ^18^F-FDG-PET or Single-Photon Emission Computed Tomography (SPECT), and 11 of them showed an abnormal metabolism with potential clinical significance, as shown in Table 3. Three cases presented with abnormal results on DaTSCAN (25, 26, 29).

### Response to Immunotherapy

Among the 154 cases with available treatment information, 123 patients received immunosuppressive or immunomodulatory treatment, as shown in Table 4. Five patients were lost to follow-up, and their response to immunotherapy remains unknown. Since there are several case series that does not contain detailed information about the treatment process, we will have to rule out these cases in regard to analyzing the response to immunotherapy. Among the remaining 70 patients, 43 only received empirical first-line therapy, and 27 received second-line therapy due to relapse or first-line therapy failure. The percentage of patients who responded to first-line immunotherapy was 55.8% (39/70), and the percentage of patients who had responded at the last follow-up was 54.4% (49/90). Only 17 patients were responsive to second-line treatment (63.0%). The most widely used immunosuppressive or immunomodulatory agent was steroids (63/91, 69.2%). Intravenous immunoglobulins (47/91, 51.6%), rituximab (27/91, 29.7%) and plasmapheresis (28/91, 30.8%) are also widely used for treating anti-IgLON5 disease.

**Table 4:** Immunomodulatory/immunosuppressant treatment and response to immunotherapy in described anti-IgLON5 patients.

**Immunomodulatory/immunosuppressant treatment**	***N* (%)**
Any Immunotherapy	123/154 (79.9)
Steroids	63/91 (69.2)
Immunoglobulins	47/91 (51.6)
Plasma Exchange	28/91 (30.8)
Rituximab	27/91 (29.7)
Cyclophosphamide	11/91 (12.1)
Azathioprine	9/91 (9.9)
Mycophenolate mofetil	8/91 (8.8)
**Response to Immunotherapy**	
Response to first-line therapy	39/70 (55.8)
Response to second-line therapy	17/27 (63.0)
Response to last follow-up	49/90 (54.4)
**Reason for second-line therapy**	
Relapse	9/23 (39.1)
Not effective	14/23 (60.9)

## Discussion

We reported a rare anti-IgLON5 case with atypical clinical features. With epileptic seizures as the chief presenting symptom, she also showed the following characteristic symptoms: sleep disorder and cognitive impairment. It is impossible to classify this case according to the previously described clinical phenotypes (2, 8). In addition, abnormal MRI alterations in our patient are equally noteworthy. Although abnormalities of the hippocampus in cranial MRI have been reported in other cases before, our findings have unique significance. Zhang et al. reported right hippocampal atrophy with low metabolism in the right hippocampus in a patient diagnosed with anti-IgLON5 disease (5). Hyperintensities were found in the bilateral hippocampus in a patient with anti-LGI1 and anti-IgLON5 antibodies detected in the serum and cerebrospinal fluid (23). However, the increased volume of the right hippocampus and hyperintensities in the fluid attenuated inversion recovery (FLAIR) sequence were first reported in our case and are highly suggestive of acute inflammatory lesions in the right hippocampus. Anti-IgLON5 disease is known as an autoantibody-mediated disorder. This suggests that the main mechanism of our case may be anti-IgLON5 antibody-mediated inflammation in the hippocampus, which caused hyperexcitability and finally led to epileptic seizures.

The comprehensive systematic review we performed suggests that the various clinical manifestations of anti-IgLON5 disease are far beyond our perception. The most commonly used classification of clinical phenotypes seems impractical since it does not include some uncommon or newly reported clinical symptoms. We came to another conclusion about the clinical features of anti-IgLON5 disease in this study, intending to expand the clinical spectrum of the disorder and to provide instruction for clinical decision-making. In addition, we confirmed the close relationship between haplotype DRB1*10:01-DQB1*05:01 and anti-IgLON5 disorder. There are no applicable diagnostic criteria or standards for probable or possible anti-IgLON5 disease. Our work may be useful to find additional ways to diagnose anti-IgLON5 disease.

After gathering all published evidence and analyzing the response of anti-IgLON5 patients to immunotherapy, we found that nearly half of the patients failed to respond to immunotherapy. Neuronal tau deposits were found in two patients diagnosed with anti-IgLON5 disease (30). Therefore, a failure to respond to immunotherapy may be attributed to the degenerative component of anti-IgLON5 disease. We did not perform subgroup analysis according to their clinical phenotypes because several cases cannot be classified by the current phenotypes. However, studies with larger sample sizes are needed to investigate the risk factors for the ineffectiveness of immunotherapy.

There are some unavoidable limitations of our study. We included a case series reported by Gaig et al. (8) that contained the unspecified data of several patients, which caused some difficulties in the analysis process. We were not able to determine the age at onset by median and inter quartile range (IQR), and we were not able to further analyze whether patients were responsive to first-adapted immunotherapy. In addition, only a small number of case series and isolated case reports were gathered and included in our systematic review, highly suggestive of publication bias. Considering the numerous published cases have mainly focused on a clinical description of the disease, the descriptions of the treatment outcomes and the laboratory findings are not detailed enough, which could lead to a loss of useful information. However, our work still provides a new perspective on anti-IgLON5 disease.

## Conclusion

In conclusion, we conducted a systematic review of the literature on all published anti-IgLON5 cases. The patients show heterogeneous clinical symptoms and laboratory findings and there is a need for a more comprehensive conclusion and a new diagnostic standard. The use of immunotherapy showed an unsatisfying result, with only half of the patients responding to the treatment. Further analysis is required to determine the factors influencing the efficacy of immunotherapy.

## Data Availability Statement

The original contributions presented in the study are included in the article/Supplementary Materials, further inquiries can be directed to the corresponding author.

## Author Contributions

LL, YF, and XZ: conceptualization and methodology. YF and XZ: literature search, data extraction, and formal analysis. LL: study supervision and revision of the manuscript. YF: drafting of the manuscript. All authors contributed to the article and approved the submitted version.

## Funding

This work was supported by the Science & Technology Department of Sichuan Province [Grant Number 2021YFS0174] and Health Commission of Sichuan Province [Grant Number 20ZD005].

## Conflict of Interest

The authors declare that the research was conducted in the absence of any commercial or financial relationships that could be construed as a potential conflict of interest.

## Publisher's Note

All claims expressed in this article are solely those of the authors and do not necessarily represent those of their affiliated organizations, or those of the publisher, the editors and the reviewers. Any product that may be evaluated in this article, or claim that may be made by its manufacturer, is not guaranteed or endorsed by the publisher.
