# Supplementary material for: Epileptic Seizures and Right-Sided Hippocampal Swelling as Presenting Symptoms of Anti-IgLON5 Disease: A Case Report and Systematic Review of the Literature
Source: Front Neurol. 2022 May 10;13:800298. doi: 10.3389/fneur.2022.800298 (PMC9127316; doi:10.3389/fneur.2022.800298)
Supplement: Supplementary file 1 [file Data_Sheet_1.docx]

**Identification of studies via databases and registers**

Records removed *before screening*:

Duplicate records removed (n = 260)

Records marked as ineligible by automation tools (n = 0)

Records removed for other reasons (n = 0)

Records identified from*:

PubMed (n = 125)

EMBASE (n= 168)

CNKI (n= 71)

WanFang (n= 98)

VIP China Science (n= 9)

Registers (n = 0)

**Identification**

Records screened

(n = 211)

Records excluded**

(n = 88)

Reports sought for retrieval

(n = 123)

Reports not retrieved

(n = 11)

**Screening**

Reports excluded:

Re-publication of duplicated cases (n = 19)

Comments or editorials on published cases (n = 12)

Narrative reviews (n = 7)

Lack of detailed clinical information (n= 9)

Reports assessed for eligibility

(n = 112)

Studies included in review

(n = 65)

Reports of included cases

(n = 161)

**Included**

*Consider, if feasible to do so, reporting the number of records identified from each database or register searched (rather than the total number across all databases/registers).

**If automation tools were used, indicate how many records were excluded by a human and how many were excluded by automation tools.

*From:*  Page MJ, McKenzie JE, Bossuyt PM, Boutron I, Hoffmann TC, Mulrow CD, et al. The PRISMA 2020 statement: an updated guideline for reporting systematic reviews. BMJ 2021;372:n71. doi: 10.1136/bmj.n71

For more information, visit: <http://www.prisma-statement.org/>
