# Supplementary material for: Epileptic Seizures and Right-Sided Hippocampal Swelling as Presenting Symptoms of Anti-IgLON5 Disease: A Case Report and Systematic Review of the Literature
Source: Front Neurol. 2022 May 10;13:800298. doi: 10.3389/fneur.2022.800298 (PMC9127316; doi:10.3389/fneur.2022.800298)
Supplement: Supplementary file 3 [file Data_Sheet_3.docx]

**Search strategy**

**Database: PubMed**

#1 "IgLON5 protein, human" OR "IgLON family member 5, human" OR "IgLON5" OR "IgLON5 disease" OR "IgLON5 encephalopathy" OR "IgLON5 syndrome" OR "IgLON5-mediated neurodegeneration" OR "Anti-IgLON5-related tauopathy" OR "Autoimmune encephalitis with anti-IgLON5" OR "IgLON5 associated encephalitis" OR "IgLON5 autoimmunity" OR "IgLON5 antibody" OR "Anti-IgLON5" OR "Autoantibodies to IgLON5"

**Database: Embase**

#1 IgLON5.mp.

#2 IgLON family member 5.mp.

#3 IgLON5 disease.mp.

#4 IgLON5 protein.mp.

#5 IgLON5 encephalopathy.mp.

#6 IgLON5 syndrome.mp.

#7 IgLON5-mediated neurodegeneration.mp.

#8 Anti-IgLON5-related tauopathy.mp.

#9 Autoimmune encephalitis with anti-IgLON5.mp.

#10 IgLON5 associated encephalitis.mp.

#11 IgLON5 autoimmunity.mp.

#12 IgLON5 antibody.mp.

#13 Anti-IgLON5.mp.

#14 Autoantibodies to IgLON5.mp.

#15 1 or 2 or 3 or 4 or 5 or 6 or 7 or 8 or 9 or 10 or 11 or 12 or 13 or 14
